# NIR luminescence lifetime nanothermometry based on phonon assisted Yb^3+^–Nd^3+^ energy transfer†

**DOI:** 10.1039/d1na00285f

**Published:** 2021-06-14

**Authors:** K. Maciejewska, A. Bednarkiewicz, L. Marciniak

**Affiliations:** Institute of Low Temperature and Structure Research, Polish Academy of Sciences Okólna 2 50-422 Wroclaw Poland l.marciniak@intibs.pl

## Abstract

Luminescence thermometry in biomedical sciences is a highly desirable, but also highly challenging and demanding technology. Numerous artifacts have been found during steady-state spectroscopy temperature quantification, such as ratiometric spectroscopy. Oppositely, the luminescence lifetime is considered as the most reliable indicator of temperature thermometry because this luminescent feature is not susceptible to sample properties or luminescence reabsorption by the nanothermometers themselves. Unfortunately, this type of thermometer is much less studied and known. Here, the thermometric properties of Yb^3+^ ions in Nd_0.5_RE_0.4_Yb_0.1_PO_4_ luminescent temperature probes were evaluated, aiming to design and optimize luminescence lifetime based nanothermometers. Temperature dependence of the luminescence lifetimes is induced by thermally activated phonon assisted energy transfer from the ^2^F_5/2_ state of Yb^3+^ ions to the ^4^F_3/2_ state of Nd^3+^ ions, which in turn is responsible for the significant quenching of the Yb^3+^:^2^F_5/2_ lifetime. It was also found that the thermal quenching and thus the relative sensitivity of the luminescent thermometer can be intentionally altered by the RE ions used (RE = Y, Lu, La, and Gd). The highest relative sensitivity was found to be *S*_R_ = 1.22% K^−1^ at 355 K for Nd_0.5_Y_0.4_Yb_0.1_PO_4_ and it remains above 1% K^−1^ up to 500 K. The high sensitivity and reliable thermometric performance of Nd_0.5_La_0.4_Yb_0.1_PO_4_ were confirmed by the high reproducibility of the temperature readout and the temperature uncertainty being as low as *δT* = 0.05 K at 383 K.

## Abstract 2

Luminescence thermometry in biomedical sciences is a highly desirable, but also highly challenging and demanding technology.

## Graphical abstract

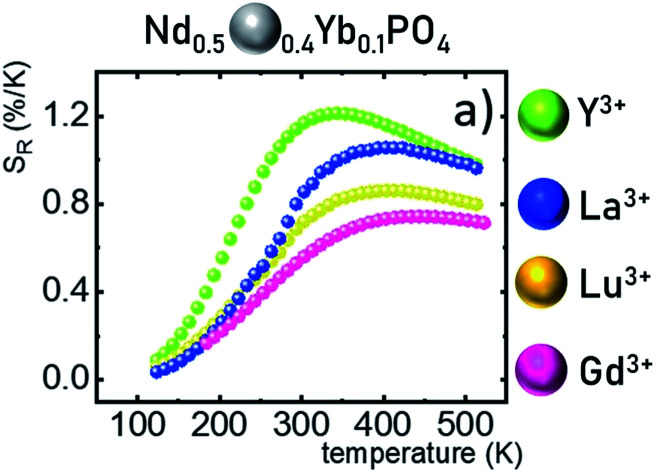

## 1. Introduction

The consistent growth of scientific interest in luminescence thermometry (LT), a technique that exploits temperature-induced changes in the luminescence properties of phosphors for temperature readout, results from the capability of remote thermal imaging that it offers.^1–7^ One of the most sophisticated examples of such remote temperature readout is *in vivo* thermal imaging of biological systems, where, in a noninvasive and electrically passive manner, information about temperature distribution in the living cells and tissues can be visualized with a submicrometer spatial resolution.^6,8–11^ This may not only provide the information about the rate of the biological processes (*e.g.* thermogenesis) but may also enable feedback-controlled medical treatments (*e.g.* hyperthermal therapy).^12–14^

The most commonly exploited spectroscopic feature of the thermometric phosphors that, after calibration, reports about temperature is the shape of luminescence spectra. Most commonly, the intensity ratio of two emission bands is used.^15–24^ However, because the shape of the emission band may be affected by the unknown and sample-to-sample variable absorption of the medium surrounding the phosphor or by interparticle interactions within the sample, the credibility of the ratiometric LT approach, especially in the *in vivo* applications, has been recently undermined.^25,26^ Although some strategies to mitigate, correct or circumvent these limitations have been recently proposed by involving a primary thermometry concept or multiparametric luminescent thermometers, these approaches require complicated correction and analysis of the obtained data.^27^

An important alternative temperature indicator utilizes temperature dependent kinetics of the excited states. Unlike short living fluorophores (*e.g.* organic dyes, fluorescent proteins, and most QDs), the luminescence lifetime of excited states of lanthanides is almost unaffected by the absorption or scattering by the medium, and when the surface of the phosphor is isolated from the surroundings by the optically passive shell, its kinetics is also independent of the local chemical environment. Despite this concept being promising, the relative sensitivity of lifetime-based LTs is usually considered lower with respect to the ratiometric counterpart.^5,28–31^ This is because the excited level kinetics is modified by quenching through temperature dependent multi-phonon processes. Therefore, to boost the sensitivity of luminescence lifetime based LTs, the other energy transfer processes susceptible to temperature should be involved.

As has been recently demonstrated, the energy transfer relying on the absorption of phonons could be an appropriate direction to follow.^32^ Therefore, in this manuscript, a comprehensive and detailed investigation on the temperature dependence of phonon mediated energy transfer between Yb^3+^ and Nd^3+^ ions is performed in order to develop a highly sensitive luminescent thermometer based on the kinetics of the Yb^3+^ ions. As the probability of phonon assisted energy transfer depends on many material parameters of the host, the performed studies have been conducted on a series of Nd_0.5_RE_0.4_Yb_0.1_PO_4_ (where RE = Y^3+^, Lu^3+^, La^3+^, and Gd^3+^) nanocrystals in order to select the most promising candidate. The proposed phosphate matrix is inherently biocompatible, resistant to strong oxidizing and reducing acids (nitric and hydrochloric acids) and enables easy fine tuning of spectral properties by intentional substitution of RE with numerous optically passive ions (*e.g.* RE: Y^3+^, Lu^3+^, La^3+^, and Gd^3+^ which differ in ionic radius, coordination number, *etc.*).^33–35^

## 2. Materials and methods

### Materials

The monazite Nd_1−*x*_RE_*x*_PO_4_:Yb^3+^ nanoparticles (NPs) (RE: Y^3+^, Lu^3+^, La^3+^, Gd^3+^) were synthesized *via* a precipitation method. All chemicals, ytterbium(iii) oxide Yb_2_O_3_ (99.99%, Alfa Aesar), europium(iii) oxide Nd_2_O_3_ (99.9%, Alfa Aesar), yttrium(iii) oxide Y_2_O_3_ (99.99%, Alfa Aesar), lutetium(iii) oxide Lu_2_O_3_ (99.99%, Alfa Aesar), lanthanum(iii) oxide La_2_O_3_ (99.99%, Alfa Aesar), gadolinium(iii) oxide Gd_2_O_3_ (99.99%, Alfa Aesar), ammonium hydrogen phosphate (NH_4_)_2_HPO_4_ (98.0%, Alfa Aesar) and polyethylene glycol (99.5%, Chempur) were used without further purification.

### Synthesis

Stoichiometric amounts of oxides (Y_2_O_3_, Lu_2_O_3_, La_2_O_3_, Gd_2_O_3_, Nd_2_O_3_ and Yb_2_O_3_) were diluted in ultrapure nitric acid to produce nitrates and placed in a Teflon-lined autoclave , followed by evaporation of the excess solution and drying over P_2_O_5_ in a vacuum desiccator for 1 day. The procedure for the synthesis of Nd_0.5_RE_0.4_Yb_0.1_PO_4_ (where RE: Y^3+^, Lu^3+^, La^3+^, and Gd^3+^) nanoparticles contains two steps. The first step consisted of the precipitation of orthophosphates in polyethylene glycol and water solution using (NH_4_)_2_HPO_4_ water solution (0.17 mol L^−1^) at 50 °C. In the second step, the slurry was centrifuged and washed three times in water and ethanol. The obtained orthophosphates were aged at 80 °C for 12 h and after that were annealed at 900 °C for 2 h to obtain the powders.

### Methods

Powder diffraction studies were carried out using a PANalytical X'Pert Pro diffractometer equipped with an Anton Paar TCU 1000 N temperature control unit using Ni-filtered Cu Kα radiation (*V* = 40 kV, *I* = 30 mA). Transmission electron microscopy images were obtained using an FEI Tecnai G2 20 X-TWIN microscope equipped with a CCD FEI Eagle 2K camera with a HAADF detector and electron gun with a LaB_6_ cathode. The pattern present on the left side of the TEM images is a microscope artifact.

An FLS1000 spectrometer from Edinburgh Instruments equipped with a R928P side window photomultiplier tube and a Hamamatsu detector was used to carry out measurements of photoluminescence decay curves and excitation spectra utilizing a micro-flash lamp and a halogen lamp, respectively. The emission spectra were measured using a Silver-Nova Super Range TEC spectrometer from Stellarnet of 1 nm spectral resolution and 808 nm excitation lines from a laser diode. The excitation pulse duration was modulated using a simple electronic PWM system. The temperature dependencies of both emission spectra and luminescence decay profiles were measured using a THMS 600 heating–cooling stage from Linkam (0.1 °C temperature stability and 0.1 °C set point resolution) to control the temperature.

## 3. Results and discussion

**Fig. 1:**
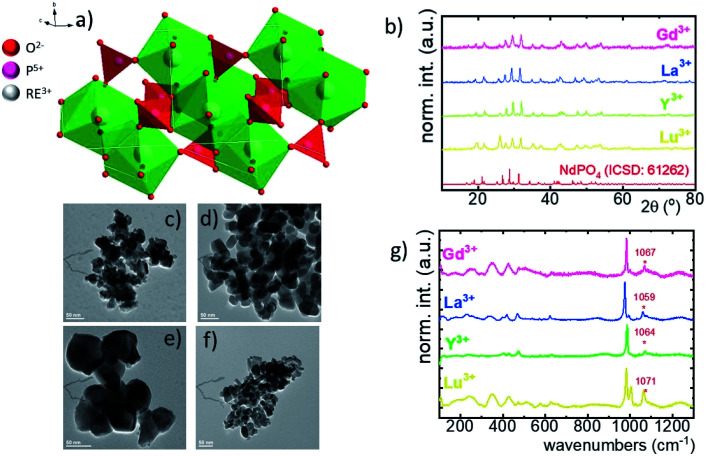
The visualization of the double cell unit of the NdPO_4_ structure (a); X-ray diffraction patterns of Nd_0.5_La_0.4_Yb_0.1_PO_4_, Nd_0.5_Y_0.4_Yb_0.1_PO_4_, Nd_0.5_Lu_0.4_Yb_0.1_PO_4_ and Nd_0.5_Gd_0.4_Yb_0.1_PO_4_ (b). The representative TEM images of Nd_0.5_La_0.4_Yb_0.1_PO_4_ (c), Nd_0.5_Y_0.4_Yb_0.1_PO_4_ (d), Nd_0.5_Lu_0.4_Yb_0.1_PO_4_ (e) and Nd_0.5_Gd_0.4_Yb_0.1_PO_4_ (f) nanocrystals; the comparison of the Raman spectra for Nd_0.5_RE_0.4_Yb_0.1_PO_4_ nanocrystals (g).

Although the RE orthophosphates crystalize in three different crystallographic structures (tetragonal, monoclinic and hexagonal) due to the ionic size of the Nd^3+^ ions, NdPO_4_ crystalizes in the monazite-type monoclinic structure of *P*2_1_/*c* (ref. 14) symmetry with the following unit parameters: *a* = 6.4537 Å, *b* = 7.0425 Å, *c* = 8.2012 Å, and *β* = 125.8328°. In this case, the [PO_4_]–[REO_*x*_]–[PO_4_] chains are interlinked with 9 oxygen ions (Fig. 1a).^36^ The nine-fold coordinated site of the Nd^3+^ ions of *C*_1_ point symmetry can be successfully substituted by the RE ions because of the similarity in the ionic radii. The phase purity of the synthesized nanocrystals has been confirmed by the powder X-ray diffraction pattern measurements. All of the observed diffraction reflexes correspond to the reference pattern (ICSD 61262). However, due to the difference in the ionic radii between the substituting RE and Nd^3+^ ions, and in consequence a change of the unit cell parameters, the shift of the diffraction peaks can be observed. The values of the cell parameters obtained from the Rietveld refinement method are listed in Table 1. It can be clearly seen that for the Nd_0.5_RE_0.4_Yb_0.1_PO_4_ nanocrystals, the cell volume increases according to the following order: *V*_Y^3+^_ < *V*_Lu^3+^_ < *V*_Gd^3+^_ < *V*_La^3+^_. As was confirmed by the TEM studies, the synthesized powders consist of the aggregated nanocrystals (Fig. 1c–f, see also Fig. S1†). The diameter of the nanocrystals is affected by the type of the RE cation used, reaching *d* = 40 ± 12 nm for Y^3+^; *d* = 55 ± 10 nm for Lu^3+^; *d* = 60 ± 10 nm for La^3+^; *d* = 32 ± 12 nm for Gd^3+^ (Fig. S2†). In the case of Raman spectra, most of the recorded bands are characteristic of the vibrations of the phosphate groups (*i.e.* P–O symmetric stretching, P–O asymmetric stretching, and O–P–O symmetric/asymmetric bending vibrations). However, all the modes are shifted toward higher energies in a sequence La^3+^ < Y^3+^ < Lu^3+^ < Gd^3+^ (dashed area in the spectra).

**Table 1:** The calculated structural parameters of Nd_0.5_RE_0.4_Yb_0.1_PO_4_ nanocrystals based on the XRD patterns

RE^3+^	Crystal structure	Point symmetry	Max phonon energy (cm^−1^)	*a*	*b*	*c*	Cell volume (Å^3^)	Ionic radius of RE (Å) [coordination number]
Lu	Monoclinic	*C*_1_	1071	6.35179	6.85549	8.01249	348.90	1.019 [8]
Y	Monoclinic	*C*_1_	1063	6.37804	6.87694	8.03117	352.26	0.977 [8]
La	Monoclinic	*C*_1_	1060	6.44380	6.98207	8.17155	367.65	1.216 [9]
Gd	Monoclinic	*C*_1_	1072	6.36476	6.88189	8.04216	352.26	1.107 [9]

**Fig. 2:**
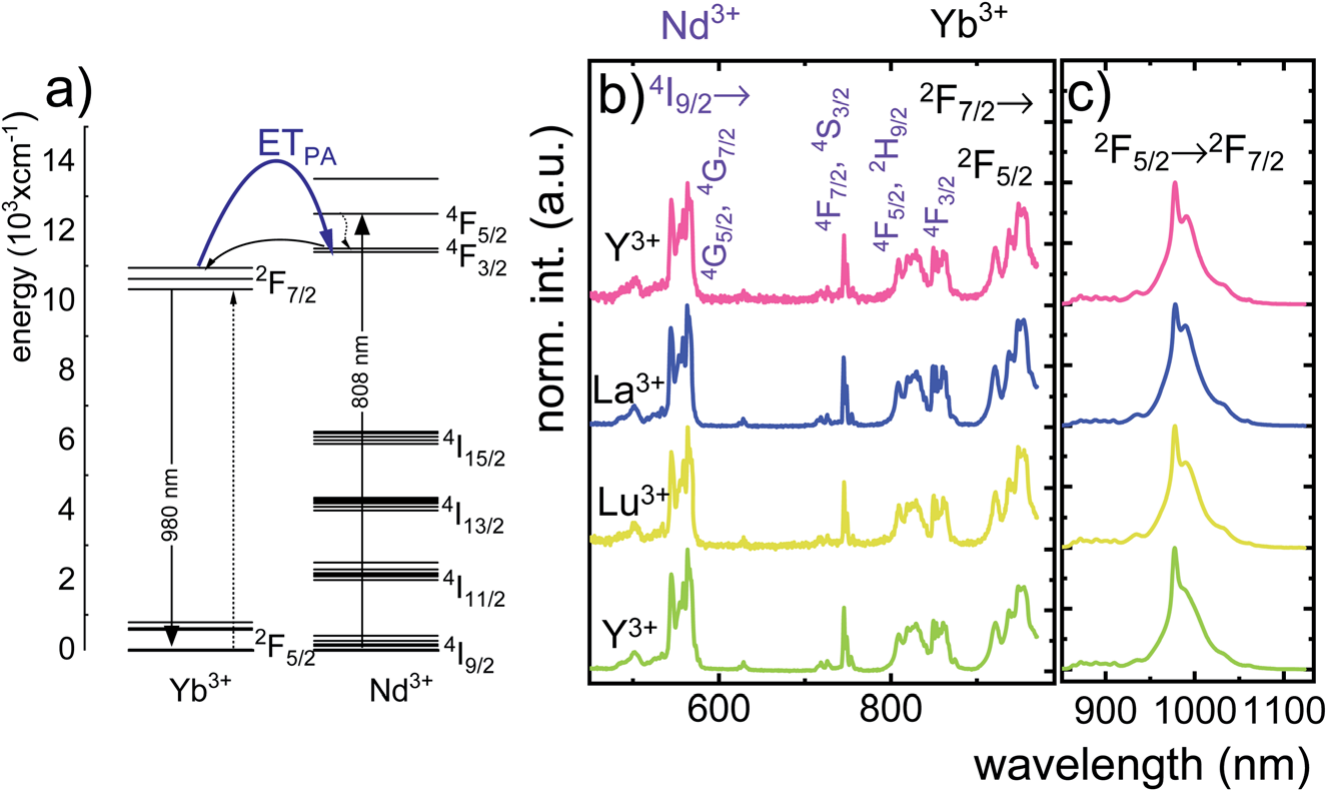
The schematic simplified energy level diagram of Yb^3+^ and Nd^3+^ ions in Nd_0.5_RE_0.4_Yb_0.1_PO_4_ (a), comparison of the normalized excitation spectra monitored at the emission of Yb^3+^ ions at *λ*_em_ = 980 nm (b) and emission spectra upon *λ*_exc_ = 808 nm (c) for different Nd_0.5_RE_0.4_Yb_0.1_PO_4_ nanocrystals.

In order to explain the luminescence properties of the Nd_0.5_Lu_0.5_PO_4_:Yb^3+^ nanocrystals, simplified energy level diagrams of Nd^3+^ and Yb^3+^ ions are presented in Fig. 2a. As can be found, the Yb^3+^ energy level scheme is relatively simple and consists only of two energy levels –the ^2^F_7/2_ ground state and ^2^F_5/2_ excited state. Therefore, upon *λ*_exc_ = 940 nm photoexcitation, which corresponds to the energy difference between these two states, only a single emission band of Yb^3+^ at *λ*_exc_ = 980 nm can be observed. The relatively large energy gap between these two states leads to the low probability of the nonradiative ^2^F_5/2_ state depopulation *via* multiphonon processes. On the other hand, the Nd^3+^ ions are characterized by the more abundant energy state configuration. The radiative depopulation of the excited metastable ^4^F_3/2_ state leads to the occurrence of three emission bands usually localized at around 880 nm, 1060 nm and 1350 nm attributed to ^4^F_3/2_ → ^4^I_9/2_, ^4^F_3/2_ → ^4^I_11/2_ and ^4^F_3/2_ → ^4^I_13/2_, respectively. The fact that the ^4^F_3/2_ energy level is localized roughly 900 cm^−1^ above that of the ^2^F_5/2_ state of Yb^3+^ ions allows for energy transfer between these ions which however must be accompanied by the absorption of the host phonon. According to the Miyakava–Dexter theorem, the probability of the interionic energy transfer *W*_ET_(*T*) is expressed using the following formula:^37–40^1
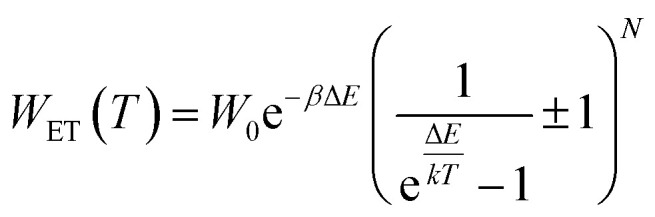
where the parameters *W*_0_, *β*, Δ*E*, *k*, *T* and *N* represent the resonant energy transfer probability between the interacting ions, a material parameter, energy gap between interacting energy levels, Boltzmann constant, temperature in Kelvin and the number of phonons involved in the process, respectively. At elevated temperatures, energy from the Yb^3+^:^2^F_5/2_ state can be transferred to the Nd^3+^:^4^F_3/2_ state with the absorption of the phonon, which will lead to the quenching of the ^2^F_5/2_ → ^2^F_7/2_ Yb^3+^ luminescence and results in intensity dimming and shortening of the Yb^3+^:^2^F_5/2_ lifetime. The opposite energy transfer from Nd^3+^ to Yb^3+^ may also occur, but in this case the emission of the phonon is more probable compared to the phonon absorption. The confirmation of the Nd^3+^ to Yb^3+^ energy transfer is the appearance of the characteristic absorption bands of Nd^3+^ associated with the 4f–4f electronic transition in the excitation spectra monitored for the emission of Yb^3+^ ions at *λ*_em_ = 980 nm (Fig. 2b). Besides the Nd^3+^ absorption band, one of the most intense absorption bands in the recorded excitation spectra is located at 940 nm which shall be associated with the ^2^F_7/2_ → ^2^F_5/2_ transition of Yb^3+^ ions. Moreover, the Nd^3+^ → Yb^3+^ energy transfer is confirmed by the presence of the Yb^3+^:^2^F_5/2_ → ^2^F_7/2_ emission band under *λ*_exc_ = 808 nm optical excitation, which corresponds to the ^4^I_9/2_ → ^4^F_5/2_, ^2^H_9/2_ electronic transition of Nd^3+^ ions. The excitation and emission spectra (Fig. 2b and c) do not reveal any significant differences for different Nd_0.5_RE_0.5_PO_4_:Yb^3+^ nanocrystals. The comparison of the integral emission intensities of the Yb^3+^ luminescence associated with the ^2^F_5/2_ → ^2^F_7/2_ electronic transition measured at room temperature reveals that Nd_0.5_Gd_0.5_PO_4_:Yb^3+^ emits two times brighter than Nd_0.5_La_0.5_PO_4_:Yb^3+^ and Nd_0.5_Y_0.5_PO_4_:Yb^3+^ and even 5 times brighter than Nd_0.5_Lu_0.5_PO_4_:Yb^3+^ (Fig. S5†).

**Fig. 3:**
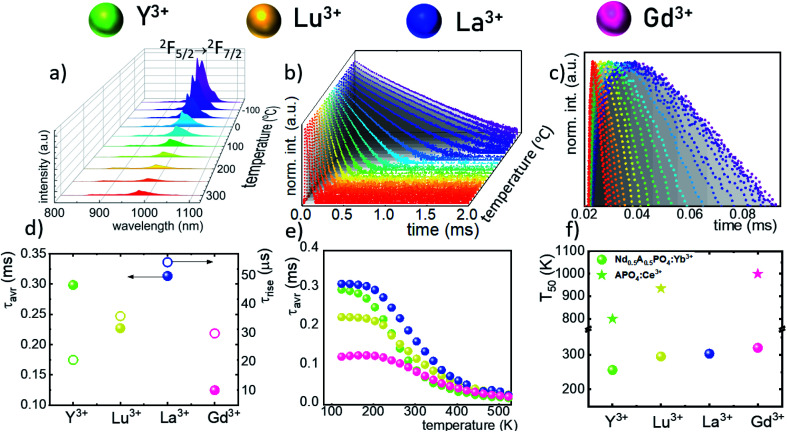
Thermal evolution of the emission spectra of Nd_0.5_Y_0.4_Yb_0.1_PO_4_ nanocrystals (a) and luminescence decay profile of the ^2^F_5/2_ state of Yb^3+^ ions (b); thermal evolution of the rise times (c); the colours on (a)–(c) correspond to each other. The comparison of the decay times and rise times measured at 110 K (d) and thermal evolution of the *τ*_avr_ (e) for different host materials; the *T*_50_ parameter for Nd_0.5_RE_0.4_Yb_0.1_PO_4_ (dots) and REPO_4_:Ce^3+^ (stars) (f).

The technical feasibility and accuracy of lifetime-based remote temperature determination can be improved when the lifetime of the emitting center is relatively long and strongly temperature dependent, respectively. The feasibility of this method is improved from efficient phosphors, which can be easily photoexcited with cheap, powerful and all-electronically controlled laser diodes, while the detection of long (μs–ms) kinetics can be easily performed with current Si based photosensors equipped with simple (opposite to digitizers of sub ns–ns lifetimes) digital oscilloscope based fluorescence acquisition. Moreover, in the case of some specific applications, such as thermal bioimaging, longer luminescence decay enables temporal separation of the luminescence of the thermal probe from the background emission (*i.e.* autofluorescence) of the tissue. Additionally, boosting the sensitivity of the thermal probe to temperature changes is possible, because the probability of the energy transfer with the absorption of the phonon (eqn (1)) is strongly dependent on temperature. Therefore, a luminescent thermometer that involves this process is expected to possess favorable thermometric properties. The probability *W*_0_ depends on the spatial distance between the interacting ions. Therefore, in order to enhance the probability of the phonon assisted energy transfer, shortening ofthe Nd^3+^ to Yb^3+^ distance is desired. As already reported by many authors, an increase of the Yb^3+^ concentration leads to the enhancement of the quenching of the Yb^3+^ luminescence *via* the migration of energy from the excited states of Yb^3+^ to the quenching centers.^41,42^ It is especially acute in the case of the nanosized phosphors for which the distance to the particle surface is relatively low. Therefore, in order to avoid the activation of this quenching channel, the concentration of Nd^3+^ ions has been increased in this work. As can be seen, initially, for low Nd^3+^ concentrations, the probability of phonon assisted energy transfer was relatively low, which is confirmed by relatively small changes of the ^2^F_5/2_ lifetime at elevated temperatures (Fig. S3†). An increase of the Nd^3+^ amount up to 50% results in significant thermal quenching of the ^2^F_5/2_ state, which results from the phonon-assisted energy transfer from Yb^3+^ to Nd^3+^ ions being facilitated by the short distance between interacting ions. These results may suggest that a further increase of the Nd^3+^ concentration should be beneficial for the thermometric performance. However, the increase of the Nd^3+^ concentration leads to the shortening of the initial lifetime value (measured at 83 K) which may hinder the luminescence kinetics measurement at higher temperatures. Therefore, in order to satisfy the conditions of long luminescence lifetime and strong temperature dependence of the luminescence lifetime of the ^2^F_5/2_ state, a concentration of 50% Nd^3+^ seems to be optimal. It must be noted here that the luminescence lifetime values themselves determine and actually limit the temporal resolution of temperature imaging in general. Therefore, although long luminescence lifetimes simplify technical means to detect and record the signal, they may concurrently limit the capabilities to image fast and dynamic processes. The phonon assisted energy transfer between Yb^3+^ and Nd^3+^ ions leads to the significant quenching of the Yb^3+^ luminescence which is reflected by the lowering of the ^2^F_5/2_ → ^2^F_7/2_ emission intensity (Fig. 3a, see also Fig. S4†), which reached 50% of its initial intensity at around 0 °C. Moreover, the shape of the emission band was slightly changed which can be associated with the thermalization of the higher lying Stark components of the ^2^F_5/2_ state at elevated temperatures. As presented in Fig. 3b, the luminescence decay profile of Yb^3+^ ions significantly changes in this temperature range (see also Fig. S6 and S7†). Notably, an in-depth analysis of the obtained decay profiles reveals the short rise-time which is associated with the Nd^3+^ → Yb^3+^ energy transfer. Due to the fact that at higher temperatures the profiles slightly deviate from the exponential decay, the average lifetime of the excited states has been calculated as follows:2
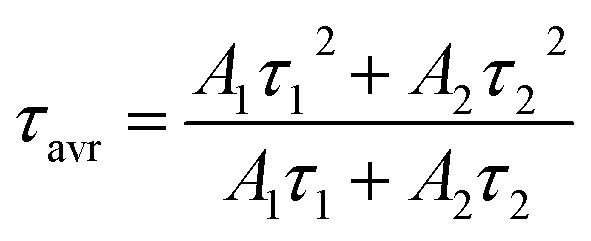


The analysis of the temperature dependence of *τ*_avr_ indicates that the RE^3+^ ions significantly affect the rate and the temperature of the activation of the thermal quenching of the ^2^F_5/2_ luminescence. The initial (at 110 K) *τ*_avr_ decreases from *τ*_avr_ = 0.31 ms for La^3+^, *τ*_avr_ = 0.29 ms for Y^3+^, *τ*_avr_ = 0.22 ms for Lu^3+^ to *τ*_avr_ = 0.12 ms for Gd^3+^. This dependence may be associated both with the interionic distance between neighboring Nd^3+^ and Yb^3+^ ions (which is dependent on the ionic radii of the RE^3+^ ions) and the phonon energy of the host material. It can also be noted that the value of the rise times is also affected by the host material composition. In the case of Nd_0.5_Y_0.4_Yb_0.1_PO_4_, when the temperature increases, thermal quenching of the ^2^F_5/2_ state instantly initializes. On the other hand, the quenching process started around 200 K for Nd_0.5_La_0.4_Yb_0.1_PO_4_ and Nd_0.5_Y_0.4_Yb_0.1_PO_4_. The highest thermal susceptibility reveals Nd_0.5_Gd_0.4_Yb_0.1_PO_4_ for which *τ*_avr_ starts to decreases above 220 K. In the case of all the host materials under investigation, the most significant *τ*_avr_ changes can be observed in the 200–400 K temperature range. In order to quantify the quenching process, the temperature at which *τ*_avr_ reached half of its initial value (*T*_50_) was considered (Fig. 3f). As can be seen, the *T*_50_ increases as follows: *T*_50_ = 256 K (Y^3+^), *T*_50_ = 295 K (Lu^3+^), *T*_50_ = 303 K (La^3+^) and *T*_50_ = 320 K (Gd^3+^). A similar trend was reported previously by Witkowski *et al.*^43^ for REPO_4_:Ce^3+^ (the stars in Fig. 3f). Although the thermal quenching process in this case was discussed in terms of electron transfer to the conduction band, a similar trend and influence of the host material was observed in the case of the Nd^3+^,Yb^3+^ co-doped systems.

**Fig. 4:**
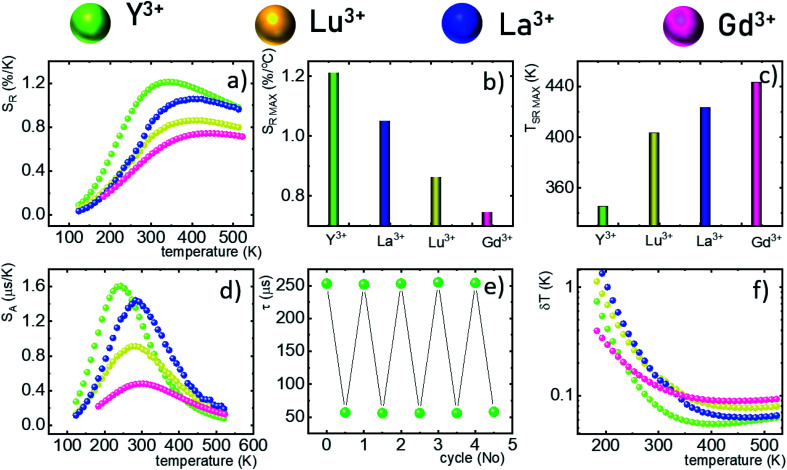
Thermal evolution of the *S*_R_ for Nd_0.5_RE_0.4_Yb_0.1_PO_4_ (a); the comparison of the *S*_RMAX_ (b) and *T*_*S*_RMAX__ (c) for different Nd_0.5_RE_0.4_Yb_0.1_PO_4_ nanocrystals; the comparison of the *S*_A_ at different temperatures (d); the lifetime of the ^2^F_5/2_ state in Nd_0.5_Y_0.4_Yb_0.1_PO_4_ in several heating–cooling cycles (e); *δT* calculated as a function of temperature (f).

One of the most important thermographic parameters that enables to directly compare the thermometric performance of different luminescent thermometers is the relative thermal sensitivity *S*_R_ which, in the case of the average luminescence lifetime used as the thermometric parameter, is defined as follows:3
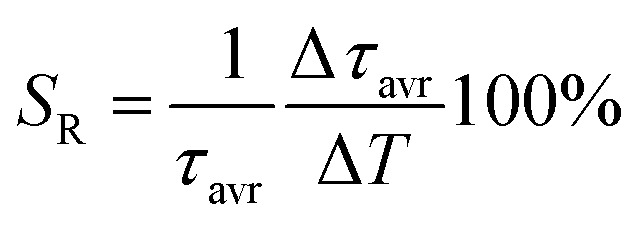
where Δ*τ*_avr_ corresponds to the change of *τ*_avr_ at Δ*T* change of temperature. In the case of the Nd_0.5_RE_0.4_Yb_0.1_PO_4_ nanocrystals, the *S*_R_ increases at elevated temperatures reaching its maximum local value above 350 K (Fig. 4a). The highest *S*_R_ = 1.22% K^−1^ was found for Nd_0.5_Y_0.4_Yb_0.1_PO_4_, while *S*_R_ = 1.05% K^−1^ for Nd_0.5_La_0.4_Yb_0.1_PO_4_, *S*_R_ = 0.85% K^−1^ for Nd_0.5_Lu_0.4_Yb_0.1_PO_4_ and *S*_R_ = 0.74% K^−1^ for Nd_0.5_Gd_0.4_Yb_0.1_PO_4_ (Fig. 4b). The temperature at which *S*_R_ reached its maximum value increases from *T*_*S*_RMAX__ = 355 K for Nd_0.5_Y_0.5_PO_4_:Yb^3+^ up to 445 K Nd_0.5_Gd_0.4_Yb_0.1_PO_4_ (Fig. 4c). It needs to be mentioned here, that despite the fact that the *S*_RMAX_ for Nd_0.5_Gd_0.4_Yb_0.1_PO_4_ was found at higher temperatures, the values of *S*_R_ for Nd_0.5_Y_0.4_Yb_0.1_PO_4_ exceed the sensitivities of the other host materials in the entire analysed temperature range. The rate of the quenching of the ^2^F_5/2_ state of Yb^3+^ ions can be also evaluated by the analysis of the absolute sensitivity (*S*_A_):4
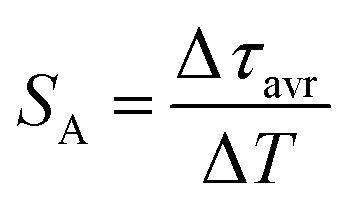


The maximum *S*_A_ = 1.65 μs K^−1^ was found for Nd_0.5_Y_0.4_Yb_0.1_PO_4_. According to the expectations, the temperature at which the maximum value of *S*_A_ was reached correlates well with the *T*_50_ temperature (Fig. 3f). It should be emphasized that in the case of the nanosized phosphors, their spectroscopic properties such as the shape of the emission spectra, luminescence lifetime and the ultimately thermometric properties may depend on the size of the nanoparticles. However, no such correlation could be found *versus* the series of passive Ln^3+^ dopants (Y^3+^, La^3+^, Gd^3+^, and Lu^3+^) used in our studies (Fig. S8†). Moreover, it was found that the *τ*_avr_ is in principle independent of the emission wavelength used, when the 950–980 nm spectral range is used (Fig. S9†). However at *λ*_em_ = 990 nm, a slightly longer value of the lifetime was found. This effect may also be related to the small difference in the crystallographic surrounding of some Yb^3+^ ions localized in the REPO_4_ nanocrystals (different crystallographic sites). This may modify the probabilities of the nonradiative depopulation rates of the ^2^F_5/2_ states, and since the contribution of the emission intensity of Yb^3+^ ions from different crystallographic sites depends on the spectral range, the lifetime may, in consequence, slightly vary with the detection wavelength used.

In order to verify the thermal stability of the synthesized nanocrystals and the repeatability of temperature readout based on the lifetime of the ^2^F_5/2_ state of the Yb^3+^ ions, the luminescence decay profiles were measured in several heating–cooling cycles (Fig. 4e). The obtained results confirm good thermometric performance of the Nd_0.5_RE_0.4_Yb_0.1_PO_4_ luminescent thermometers. Additionally the temperature determination uncertainty (*δT*) was calculated as follows:5
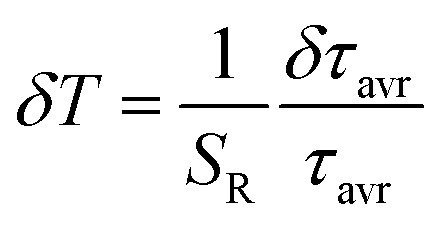
where *δτ*_avr_ represents the uncertainty of the lifetime determination (in our case, this parameter was determined as a standard deviation from 10 luminescence decay profile measurements at constant temperature). In the case of Nd_0.5_RE_0.4_Yb_0.1_PO_4_ above 350 K, all host materials revealed superior thermometric performance with the *δT* below 0.1 K. However, for Nd_0.5_Y_0.4_Yb_0.1_PO_4_, the *δT* < 0.1 K was obtained for temperatures above 273 K with the minimal uncertainty *δT* = 0.05 K at 383 K. To determine whether the duration of the excitation pulse affects the *τ*_avr_, the luminescence decay profiles of Nd_0.5_Y_0.4_Yb_0.1_PO_4_ nanocrystals were measured as a function of pulse width modulation (Fig. S10†). However no pulse duration effect was found in this case. The obtained results confirm that Nd_0.5_Y_0.4_Yb_0.1_PO_4_ exhibits the most favourable kinetics based thermometric performance among all the studied materials. It needs to be noted that the additional nonradiative quenching processes associated with ligands attached to the nanoparticle surface may reduce the emission intensity and reduce the susceptibility of the Yb^3+^ lifetimes to temperature variations. Therefore, as recently proposed by Chen *et al.*,^32^ the passivation of the nanoparticle surface by an optically inactive shell layer may improve the thermometric properties of such thermographic phosphors. Thus a relative sensitivity of 1.4% K^−1^ at 283 K was achieved for NaYF_4_@NaYF_4_:Yb^3+^,Nd^3+^@CaF_2_.^32^

## 4. Conclusions

The thermometric performance of the Yb^3+^ luminescence lifetime based thermometers in Nd_0.5_RE_0.4_Yb_0.1_PO_4_ nanocrystals was investigated in a wide temperature range. Temperature dependent phonon assisted energy transfer from the ^2^F_5/2_ excited state of Yb^3+^ to the ^4^F_3/2_ state of Nd^3+^ ions was proposed as the thermal sensitivity mechanism. The influence of temperature on the *τ*_avr_ was investigated for different RE ions (RE = Y, Lu, La, and Gd). It was found that for the optimal concentration of 50% Nd^3+^ ions at elevated temperatures, the luminescence lifetime of the Yb^3+^:^2^F_5/2_ state was rapidly quenched. The determined temperature, at which the luminescence lifetime reached half of its initial value (*T*_50_), increases from *T*_50_ = 256 K (Y^3+^) through *T*_50_ = 295 K (Lu^3+^), *T*_50_ = 303 K (La^3+^) to *T*_50_ = 320 K for Gd^3+^ passive co-dopants. High thermal quenching of the thermometric parameter (*i.e.* the lifetime of the Yb^3+^:^2^F_5/2_ state) is reflected in the thermometric performance of the described remote temperature probe. The highest absolute and relative sensitivities were found for the Nd_0.5_Y_0.4_Yb_0.1_PO_4_ compound – *i.e. S*_A_ = 1.65 μs K^−1^ at 225 K and *S*_R_ = 1.22% K^−1^ at 355 K were obtained, respectively. The change of the RE ions decreases the maximum value of the *S*_R_ and increases the temperature at which *S*_RMAX_ is reached. High thermometric performance of the Nd_0.5_Y_0.4_Yb_0.1_PO_4_ phosphor was also confirmed by good repeatability of the temperature readout within many heating–cooling cycles and by low temperature determination uncertainty (*δT* = 0.05 K at 383 K). The obtained results indicate that the phonon assisted energy transfer should be considered in further studies as a promising mechanism that may boost the sensitivity of lifetime based remote temperature probes.

## Conflicts of interest

There are no conflicts to declare.

## Supplementary Material

NA-003-D1NA00285F-s001

## References

[cit1] Dramićanin M. , in Woodhead Publishing Series in Electronic and Optical Materials, ed. M. B. T.-L. T. Dramićanin, Woodhead Publishing, 2018, pp. 1–12

[cit2] Zhou J., del Rosal B., Jaque D., Uchiyama S., Jin D. (2020). Nat. Methods.

[cit3] Bednarkiewicz A., Marciniak L., Carlos L. D., Jaque D. (2020). Nanoscale.

[cit4] Wang X., Wolfbeis O. S., Meier R. J. (2013). Chem. Soc. Rev..

[cit5] Carlos L. D. and Palacio F., Thermometry at the Nanoscale, The Royal Society of Chemistry, 2016

[cit6] Jaque D., Vetrone F. (2012). Nanoscale.

[cit7] Suta M., Meijerink A. (2020). Adv. Theory Simul..

[cit8] Brites C. D. S., Millán A., Carlos L. D. (2016). Handb. Phys. Chem. Rare Earths.

[cit9] Chrétien D., Bénit P., Ha H.-H., Keipert S., El-Khoury R., Chang Y.-T., Jastroch M., Jacobs H. T., Rustin P., Rak M. (2018). PLoS Biol..

[cit10] Arai S., Suzuki M., Park S.-J., Yoo J. S., Wang L., Kang N.-Y., Ha H.-H., Chang Y.-T. (2015). Chem. Commun..

[cit11] Kiyonaka S., Sakaguchi R., Hamachi I., Morii T., Yoshizaki T., Mori Y. (2015). Nat. Methods.

[cit12] Carrasco E., del Rosal B., Sanz-Rodríguez F., de la Fuente Á. J., Gonzalez P. H., Rocha U., Kumar K. U., Jacinto C., Solé J. G., Jaque D. (2015). Adv. Funct. Mater..

[cit13] Hemmer E., Acosta-Mora P., Méndez-Ramos J., Fischer S. (2017). J. Mater. Chem. B.

[cit14] del Rosal B., Carrasco E., Ren F., Benayas A., Vetrone F., Sanz-Rodríguez F., Ma D., Juarranz Á., Jaque D. (2016). Adv. Funct. Mater..

[cit15] Dramićanin M. and Windows B., Biomedical Applications of Luminescence Thermometry, 2018

[cit16] Dramićanin M. D. , Luminescence Thermometry, Elsevier, 2018

[cit17] Sekulić M., Đorđević V., Ristić Z., Medić M., Dramićanin M. D. (2018). Adv. Opt. Mater..

[cit18] Suta M., Antić Ž., Đorđević V., Kuzman S., Dramićanin M. D., Meijerink A. (2020). Nanomaterials.

[cit19] Marciniak L., Bednarkiewicz A., Kowalska D., Strek W. (2016). J. Mater. Chem. C.

[cit20] Maciejewska K., Marciniak L. (2020). Chem. Eng. J..

[cit21] Kniec K., Tikhomirov M., Pozniak B., Ledwa K., Marciniak L. (2020). Nanomaterials.

[cit22] Matuszewska C., Elzbieciak-Piecka K., Marciniak L. (2019). J. Phys. Chem. C.

[cit23] Kaczmarek A. M., Kaczmarek M. K., Van Deun R. (2019). Nanoscale.

[cit24] Savchuk O. A., Carvajal J. J., Brites C. D. S., Carlos L. D., Aguilo M., Diaz F. (2018). Nanoscale.

[cit25] Labrador-Páez L., Pedroni M., Speghini A., García-Solé J., Haro-González P., Jaque D. (2018). Nanoscale.

[cit26] Shen Y., Lifante J., Fernández N., Jaque D., Ximendes E. (2020). ACS Nano.

[cit27] Shen Y., Santos H. D. A., Ximendes E. C., Lifante J., Sanz-Portilla A., Monge L., Fernández N., Chaves-Coira I., Jacinto C., Brites C. D. S., Carlos L. D., Benayas A., Iglesias-de la Cruz M. C., Jaque D. (2020). Adv. Funct. Mater..

[cit28] Elzbieciak-Piecka K., Drabik J., Jaque D., Marciniak L. (2020). Phys. Chem. Chem. Phys..

[cit29] Arai S., Lee S., Zhai D., Suzuki M., Chang Y. T. (2014). Sci. Rep..

[cit30] Marciniak L., Elzbieciak-Piecka K., Kniec K., Bednarkiewicz A. (2020). Chem. Eng. J..

[cit31] Chambers M. D., Clarke D. R. (2009). Annu. Rev. Mater. Res..

[cit32] Tan M., Li F., Cao N., Li H., Wang X., Zhang C., Jaque D., Chen G. (2020). Small.

[cit33] Maciejewska K., Poźniak B., Tikhomirov M., Kobylińska A., Marciniak L. (2020). Nanomaterials.

[cit34] Runowski M., Shyichuk A., Tymiński A., Grzyb T., Lavín V., Lis S. (2018). ACS Appl. Mater. Interfaces.

[cit35] Riwotzki K., Meyssamy H., Kornowski A., Haase M. (2000). J. Phys. Chem. B.

[cit36] Milligan W. O., Mullica D. F., Beall G. W., Boatner L. A. (1982). Inorg. Chim. Acta.

[cit37] Miyakawa T., Dexter D. L. (1970). Phys. Rev. B: Solid State.

[cit38] González-Pérez S., Martín I. R., Rivera-López F., Lahoz F. (2007). J. Non-Cryst. Solids.

[cit39] Ramirez M. O., Jaque D., Bausá L. E., Martín I. R., Lahoz F., Cavalli E., Speghini A., Bettinelli M. (2005). J. Appl. Phys..

[cit40] Marciniak Ł., Bednarkiewicz A., Stefanski M., Tomala R., Hreniak D., Strek W. (2015). Phys. Chem. Chem. Phys..

[cit41] Rabouw F. T., Prins P. T., Villanueva-Delgado P., Castelijns M., Geitenbeek R. G., Meijerink A. (2018). ACS Nano.

[cit42] Pilch A., Würth C., Kaiser M., Wawrzyńczyk D., Kurnatowska M., Arabasz S., Prorok K., Samoć M., Strek W., Resch-Genger U., Bednarkiewicz A. (2017). Small.

[cit43] Witkowski D., Rothamer D. A. (2017). Appl. Phys. B: Lasers Opt..

