# Supplementary material for: NIR luminescence lifetime nanothermometry based on phonon assisted Yb3+–Nd3+ energy transfer
Source: Nanoscale Adv. 2021 Jun 14;3(17):4918–25. doi: 10.1039/d1na00285f (PMC9418974; doi:10.1039/d1na00285f)
Supplement: NA-003-D1NA00285F-s001 [file NA-003-D1NA00285F-s001.pdf]

Electronic Supplementary Material (ESI) for Nanoscale Advances.
This journal is © The Royal Society of Chemistry 2021

Supporting Information

NIR Luminescence lifetime nanothermometry based on phonon assisted Yb^{3+}-Nd^{3+} energy transfer

K. Maciejewska[1], A. Bednarkiewicz[1], L. Marciniak[1]

[1]Institute of Low Temperature and Structure Research, Polish Academy of Sciences, Okólna 2, 50-422 Wroclaw, Poland

Corresponding author: l.marciniak@intibs.pl

Keywords: luminescence thermometry, lifetimes, phonon assisted energy transfer, lanthanides, relative sensitivity, orthophosphates, nanocrystals

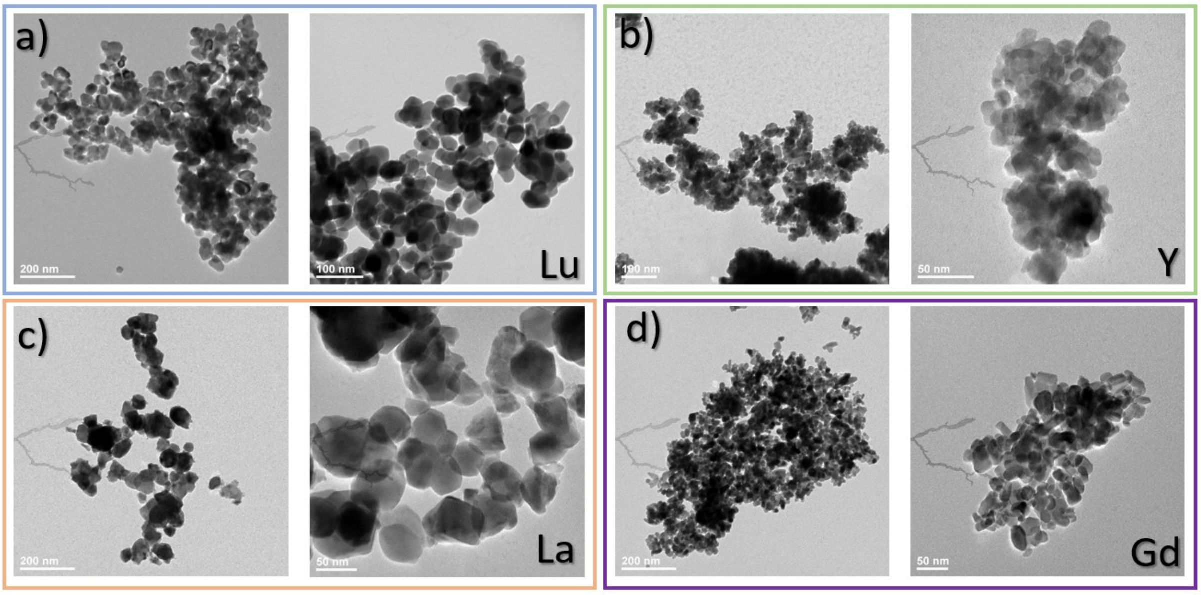

Figure S1. The representative TEM images of $Nd_{0.5}Lu_{0.4}Yb_{0.1}PO_4$ -a) $Nd_{0.5}Y_{0.4}Yb_{0.1}PO_4$ -b) $Nd_{0.5}La_{0.4}Yb_{0.1}PO_4$ -c) and $Nd_{0.5}Gd_{0.4}Yb_{0.1}PO_4$ -d).

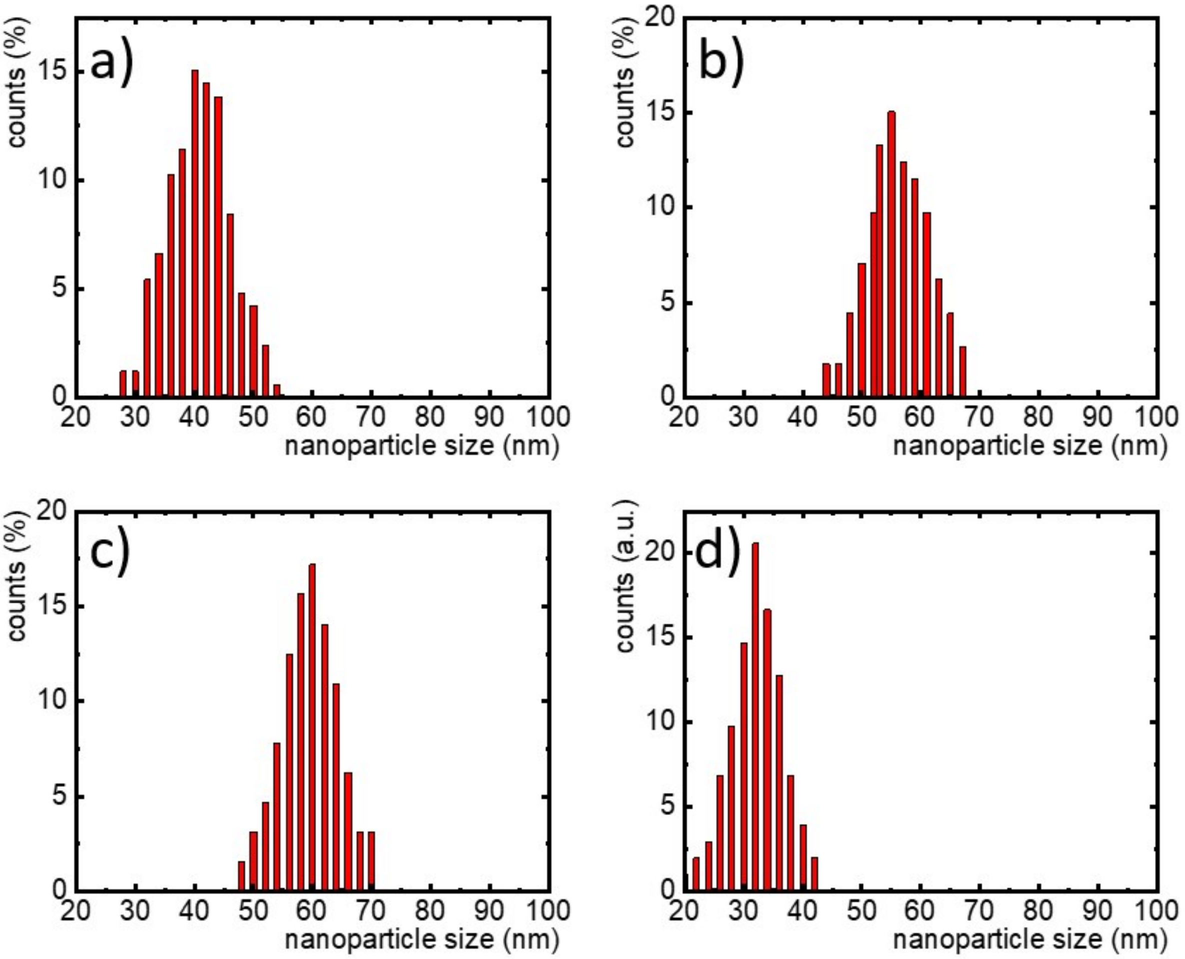

Figure S2. The histograms of the nanoparticles diameter of $Nd_{0.5}Y_{0.4}Yb_{0.1}PO_4$ -a), $Nd_{0.5}Lu_{0.4}Yb_{0.1}PO_4$ -b) $Nd_{0.5}La_{0.4}Yb_{0.1}PO_4$ -c) and $Nd_{0.5}Gd_{0.4}Yb_{0.1}PO_4$ -d).

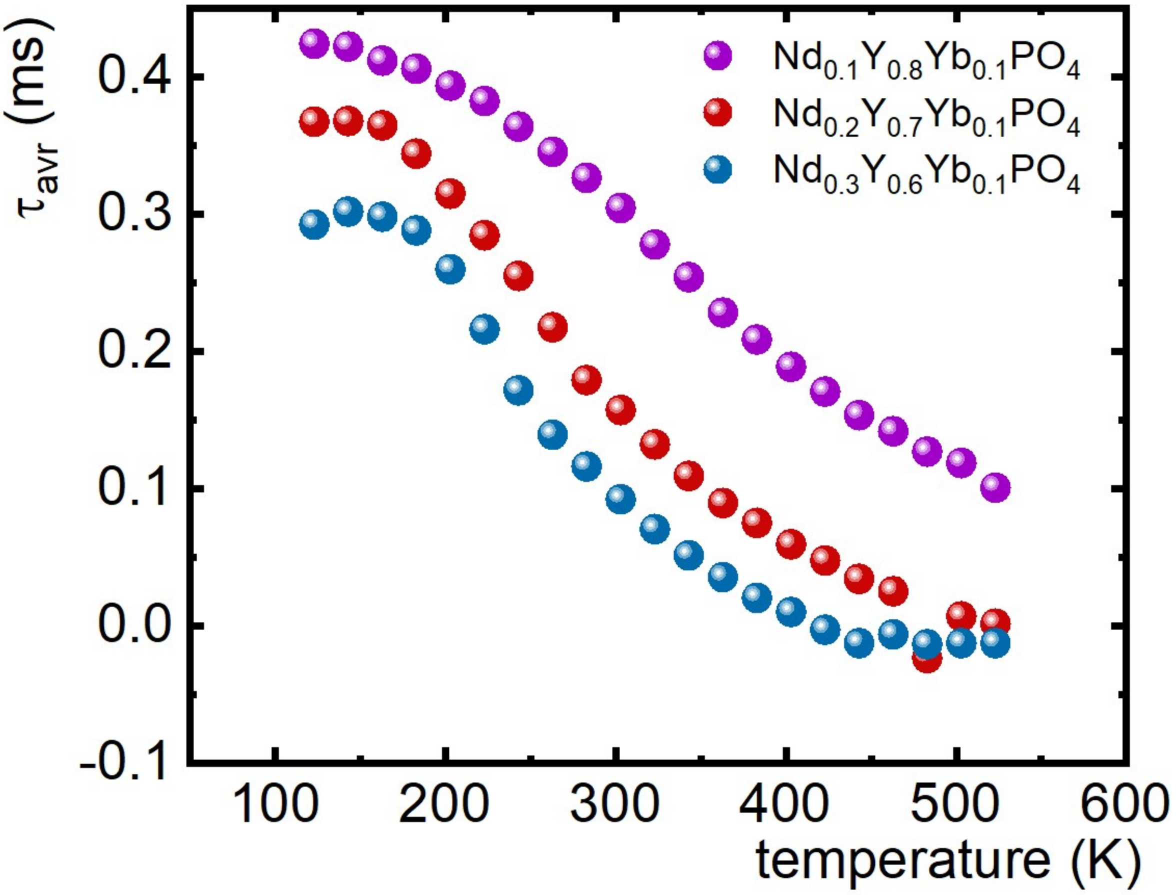

Figure S3. The τ_{avr} as a function of temperature for different concentration of Nd^{3+} ions

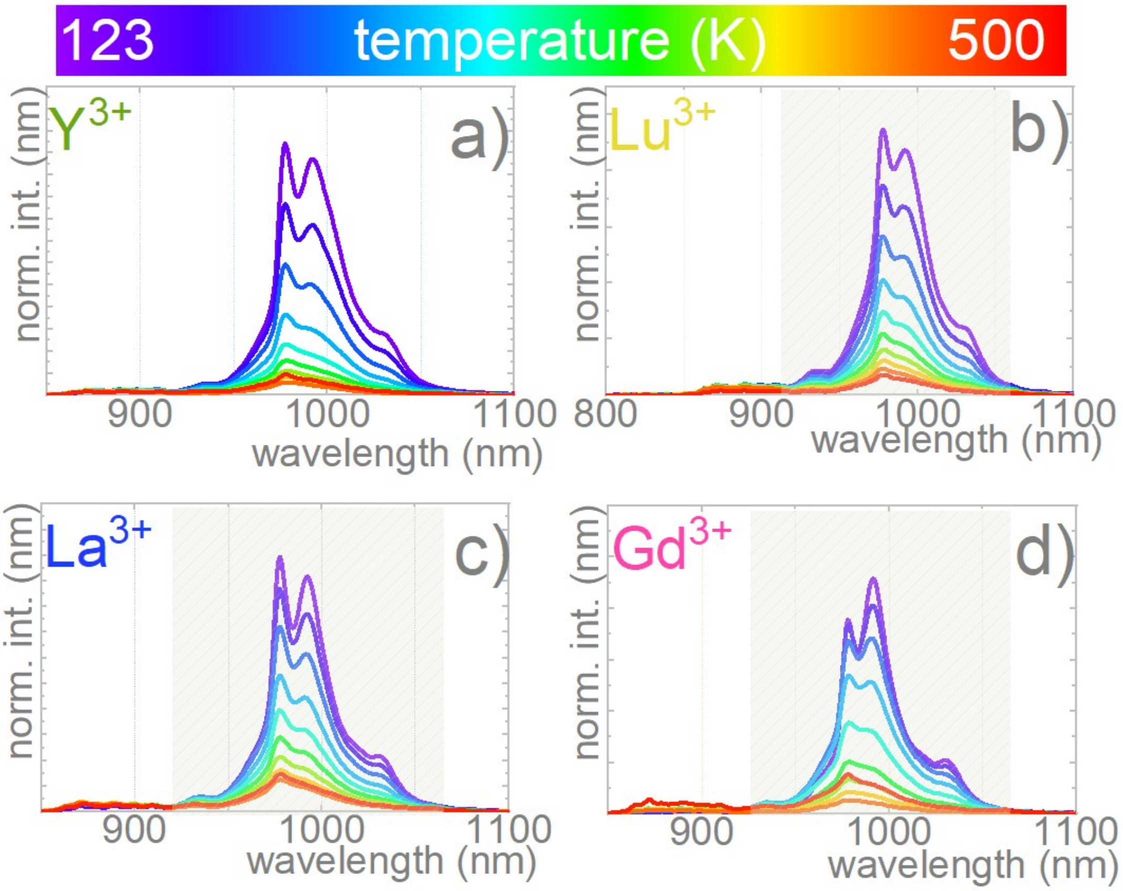

Figure S4. The emission spectra of $Nd_{0.5}Y_{0.4}Yb_{0.1}PO_4$ -a), $Nd_{0.5}Lu_{0.4}Yb_{0.1}PO_4$ -b), $Nd_{0.5}La_{0.4}Yb_{0.1}PO_4$ -c), $Nd_{0.5}Gd_{0.4}Yb_{0.1}PO_4$ -d) nanocrystals obtained upon λ_{exc}=808 nm at different temperatures.

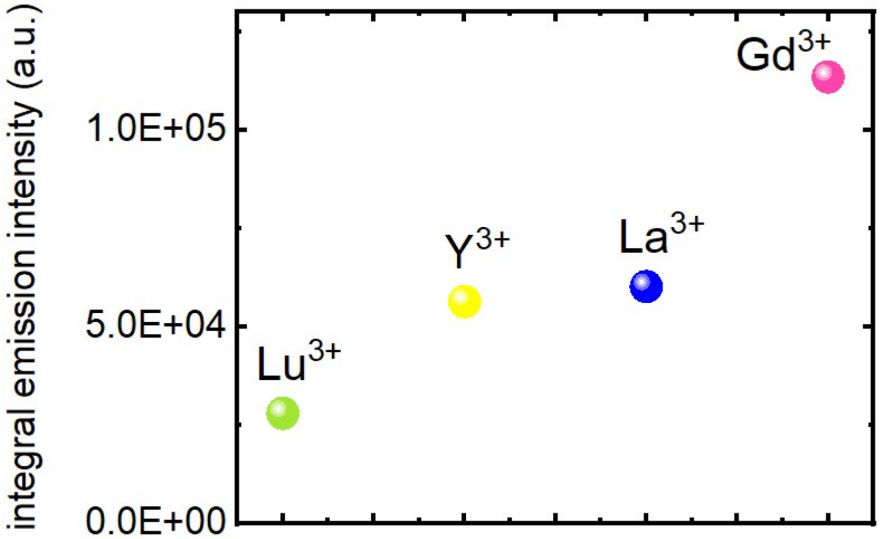

Figure S5. The comparison of the integral emission intensity of Yb^{3+} ions ($^2F_{5/2}\rightarrow{}^2F_{7/2}$ electronic transition) measured at room temperature upon λ_{exc}=808 nm

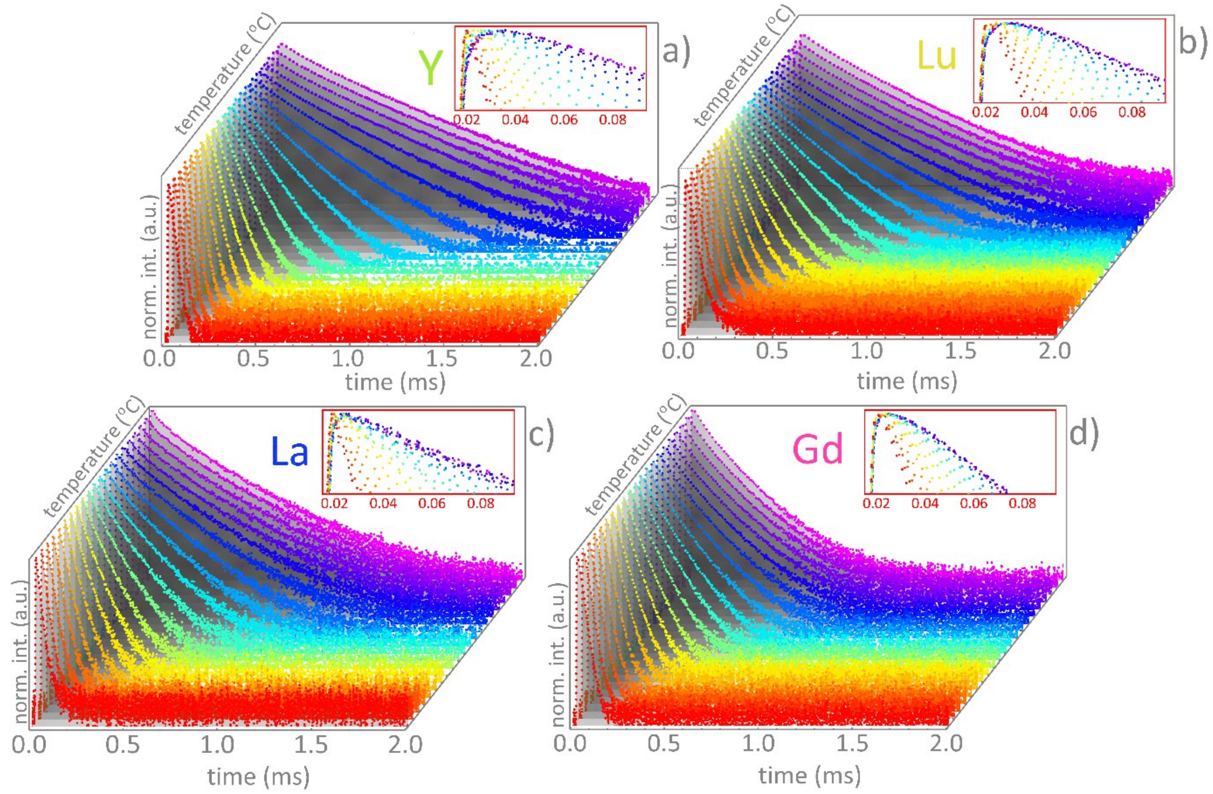

Figure S6. The luminescence decay profiles of $^2F_{5/2}$ state of Yb^{3+} ions measured at different temperatures of $Nd_{0.5}Y_{0.4}Yb_{0.1}PO_4$ -a), $Nd_{0.5}Lu_{0.4}Yb_{0.1}PO_4$ -b), $Nd_{0.5}La_{0.4}Yb_{0.1}PO_4$ -c), $Nd_{0.5}Gd_{0.4}Yb_{0.1}PO_4$ -d) nanocrystals.

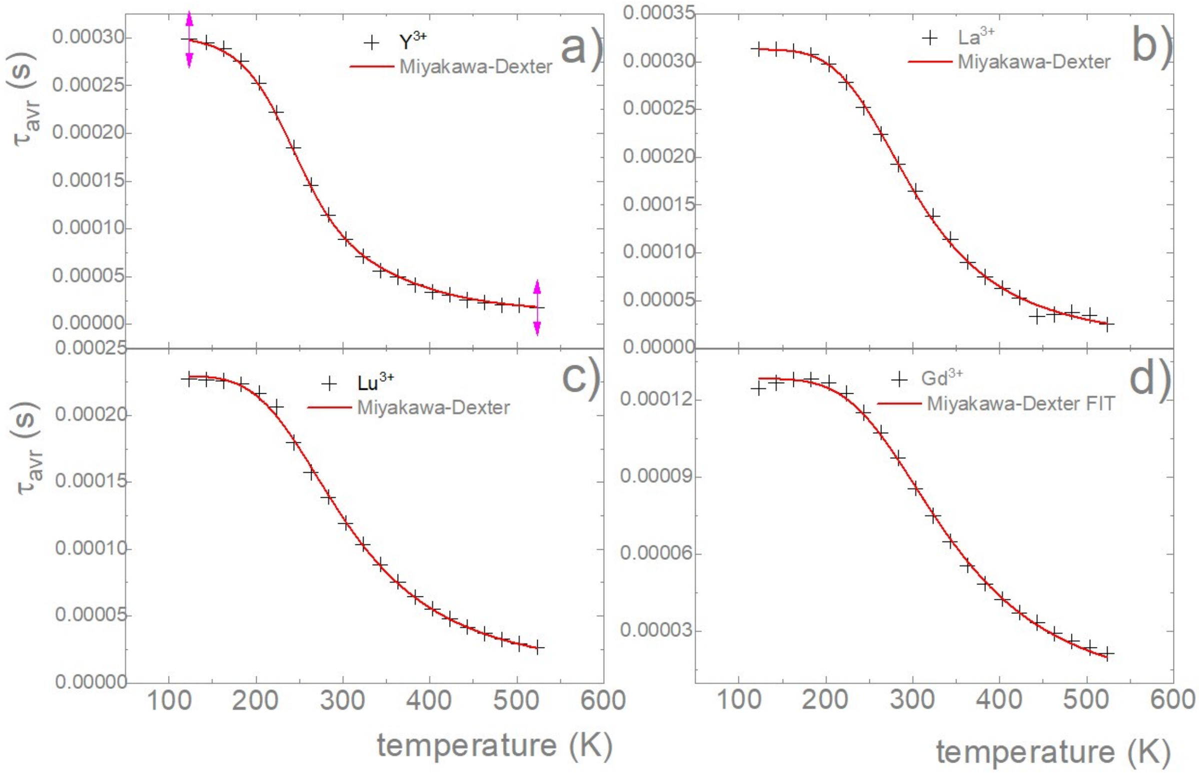

Figure S7. The τ_{avr} as a function of temperature with the fit using Miyakawa-Dexter for $Nd_{0.5}Y_{0.4}Yb_{0.1}PO_4$ -a), $Nd_{0.5}La_{0.4}Yb_{0.1}PO_4$ -b), $Nd_{0.5}Lu_{0.4}Yb_{0.1}PO_4$ -c), $Nd_{0.5}Gd_{0.4}Yb_{0.1}PO_4$ -d).

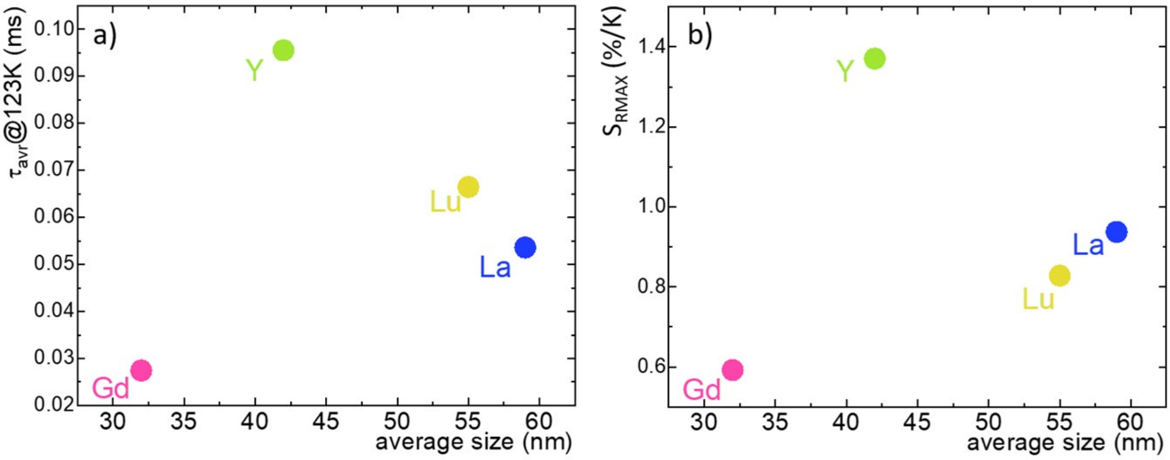

Figure S8. The τ_{avr} of the $^2F_{5/2}$ state measured at 123 K of the Yb^{3+} ions (a); and the S_{RMAX} (b) in the $REPO_4$:Nd^{3+},Yb^{3+} nanocrystals shown as a function of the average particle size.

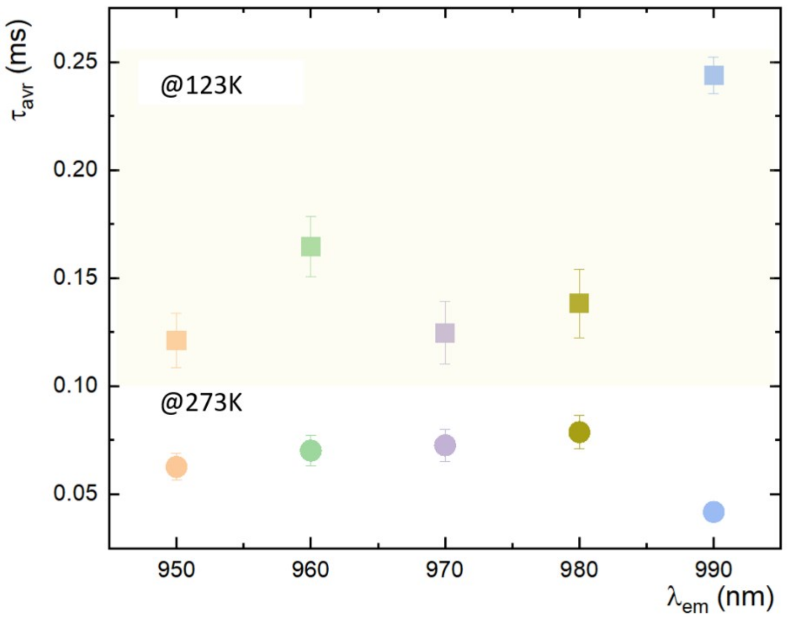

Figure S9. The τ_{avr} measured for $Nd_{0.5}Y_{0.4}Yb_{0.1}PO_4$ nanocrystals at 123 K and 273 K for different emission wavelength (λ_{em}).

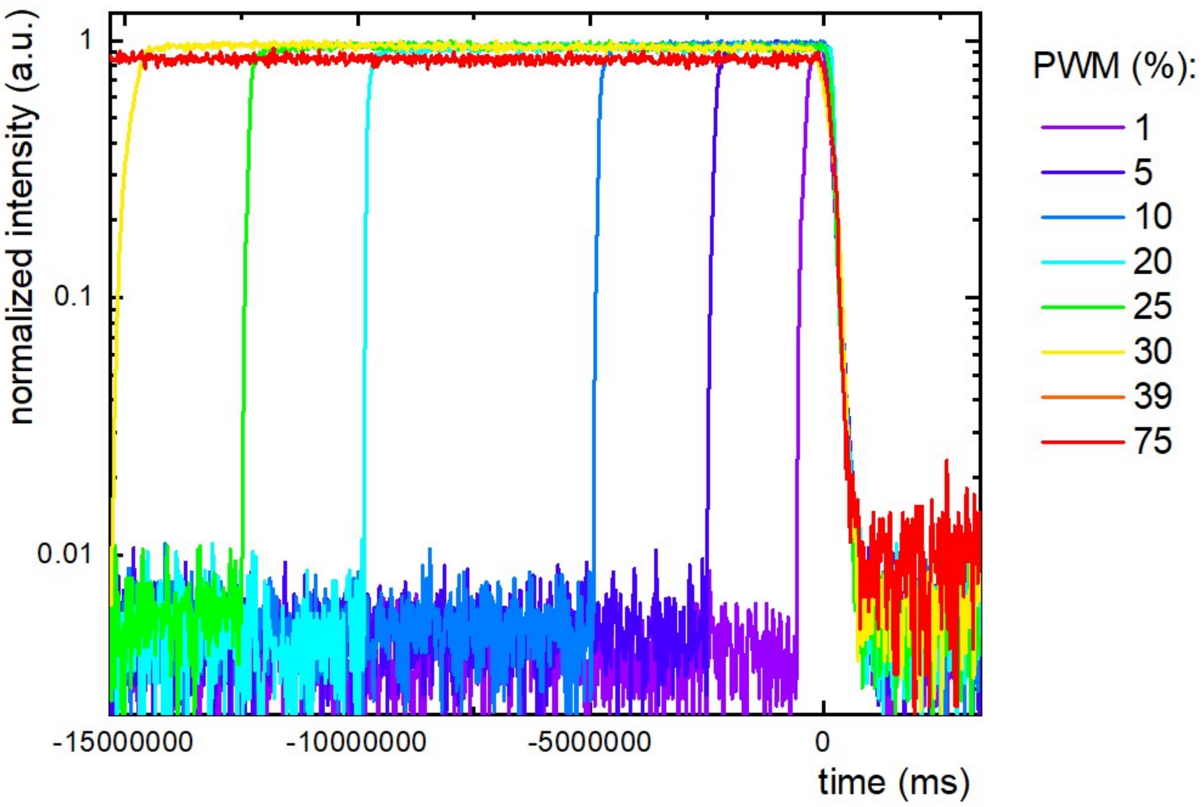

Figure S10. The comparison of the luminescence decay profile measured at 123 K for the $Nd_{0.5}Y_{0.4}Yb_{0.1}PO_4$ nanocrystals with different filling factor of pulse width modulation (PWM) λ_{exc}=808 nm, λ_{em}=980 nm.
